# Artificial Intelligence-Assisted Image Analysis of Acetaminophen-Induced Acute Hepatic Injury in Sprague-Dawley Rats

**DOI:** 10.3390/diagnostics12061478

**Published:** 2022-06-16

**Authors:** Eun Bok Baek, Ji-Hee Hwang, Heejin Park, Byoung-Seok Lee, Hwa-Young Son, Yong-Bum Kim, Sang-Yeop Jun, Jun Her, Jaeku Lee, Jae-Woo Cho

**Affiliations:** 1 College of Veterinary Medicine, Chungnam National University, Daejeon 34134, Korea; baekeunbok@hanmail.net (E.B.B.); hyson@cnu.ac.kr (H.-Y.S.); 2 Toxicologic Pathology Research Group, Department of Advanced Toxicology Research, Korea Institute of Toxicology, Daejeon 34114, Korea; jihee.hwang@kitox.re.kr (J.-H.H.); hpark@kitox.re.kr (H.P.); bslee@kitox.re.kr (B.-S.L.); 3 Department of Advanced Toxicology Research, Korea Institute of Toxicology, Daejeon 34114, Korea; ybkim@kitox.re.kr; 4 Research & Development Team, LAC Inc., Seoul 07807, Korea; syjun@lacin.co.kr (S.-Y.J.); hjun@lacin.co.kr (J.H.); jklee@lacin.co.kr (J.L.)

**Keywords:** drug-induced liver injury, acute hepatic injury, deep neural network, mask region-based convolutional neural network, artificial intelligence, deep learning

## Abstract

Although drug-induced liver injury (DILI) is a major target of the pharmaceutical industry, we currently lack an efficient model for evaluating liver toxicity in the early stage of its development. Recent progress in artificial intelligence-based deep learning technology promises to improve the accuracy and robustness of current toxicity prediction models. Mask region-based CNN (Mask R-CNN) is a detection-based segmentation model that has been used for developing algorithms. In the present study, we applied a Mask R-CNN algorithm to detect and predict acute hepatic injury lesions induced by acetaminophen (APAP) in Sprague-Dawley rats. To accomplish this, we trained, validated, and tested the model for various hepatic lesions, including necrosis, inflammation, infiltration, and portal triad. We confirmed the model performance at the whole-slide image (WSI) level. The training, validating, and testing processes, which were performed using tile images, yielded an overall model accuracy of 96.44%. For confirmation, we compared the model’s predictions for 25 WSIs at 20× magnification with annotated lesion areas determined by an accredited toxicologic pathologist. In individual WSIs, the expert-annotated lesion areas of necrosis, inflammation, and infiltration tended to be comparable with the values predicted by the algorithm. The overall predictions showed a high correlation with the annotated area. The R square values were 0.9953, 0.9610, and 0.9445 for necrosis, inflammation plus infiltration, and portal triad, respectively. The present study shows that the Mask R-CNN algorithm is a useful tool for detecting and predicting hepatic lesions in non-clinical studies. This new algorithm might be widely useful for predicting liver lesions in non-clinical and clinical settings.

## 1. Introduction

In recent years, artificial intelligence (AI)-assisted digital pathology has made rapid progress owing to the success of deep learning [1,2]. Some trials have applied deep-learning techniques in clinical and non-clinical fields of digital pathology, as they may be used to accomplish tasks that could not be automated using classical imaging analysis methods [3,4]. Deep-learning-based techniques are being increasingly applied in many routine contexts [5]; in research, they have been used in toxicological pathology and good laboratory practice (GLP) settings [6].

For digital pathology, convolutional neural networks (CNNs) are applied to build decision-making workflows [7]. When provided with plentiful data on annotated training images, CNNs can derive complex histological patterns by deconvoluting the image content into thousands of salient features, selecting/aggregating the most meaningful features, and then moving on to recognize the identified patterns in novel images [7]. Mask region-based CNN (Mask R-CNN), which was developed from Faster R-CNN, is one of the best-known detection-based segmentation models [8,9]. In Mask R-CNN, region of interest (ROI) alignment is used to increase the number of anchors and mask branches to achieve instance segmentation. Mask R-CNN has a faster detection speed and greater accuracy than Faster R-CNN [10]. To date, Mask R-CNN-based approaches have been used to analyze multiple organs, such as for heart, right-lung, and left-lung segmentation [9].

Acute hepatic injury can be caused by viral infection, alcohol, and/or drugs. The latter injury is termed drug-induced liver injury (DILI) [11,12,13]. DILI is a major concern for drug developers, regulatory authorities, and clinicians. However, we currently lack an adequate model system for assessing drug-associated DILI risk in humans [14]. The observable morphological patterns of acute hepatocellular injury include acute hepatitis, necrosis, and resolving hepatitis. Acute hepatitis is characterized by portal and parenchymal inflammation, hepatocellular injury, and/or necrosis, in the absence of fibrosis. The necrosis can be spotty or confluent; in some cases, such as that induced by acetaminophen (APAP), it can be zonal [15]. The pathological findings characteristic of APAP overdose, which include acute hepatitis with apparent centrilobular hepatic necrosis, have been targeted to develop therapeutic pharmaceuticals [16]. Several published reports have used deep learning models to predict liver injury or toxicity [17,18,19,20,21]. However, no previous study has applied deep learning to detect acute hepatic injury for toxicological diagnosis in a non-clinical study.

Here, we applied a deep-learning algorithm in developing a more efficient diagnostic tool for toxicity screening, based on the pathological characteristics of APAP-induced acute hepatic injury. We applied a Mask R-CNN segmentation network to detect the lesions of acute hepatitis, with a particular focus on lymphocyte/histiocyte infiltration and necrosis. We evaluated model performance by comparing the whole-slide image (WSI)-level detection of lesions by the model versus the annotation results generated by an accredited toxicologic pathologist.

## 2. Materials and Methods

### 2.1. Animal Experiments

Sprague-Dawley (SD) rats (Crl:CD; 9 weeks of age, both males and females) were obtained from Orient Bio, Inc. (Republic of Korea) and allowed to acclimate for 2 days prior to the beginning of the study. Throughout the experiments, the rats were maintained under controlled conditions (23 ± 3 °C, 30–70% relative humidity, 12 h light/12 h dark cycle of 150–300 lux, 10–20 cycles/h ventilation). A standard rat pellet diet (gamma-ray irradiated; 5053 PMI Nutrition International, San Francisco, CA, USA) was provided ad libitum. The animals had free access to municipal tap water that had been filtered and UV-irradiated. This water was analyzed for specific contaminants every 6 months by the Daejeon Regional Institute of Health and Environment (407, Daehak-ro, Yuseong-gu, Daejeon, Korea). The experiment was approved by the Assessment and Accreditation of Laboratory Animal Care International (AAALAC) and Institutional Animal Care and Use Committee (IACUC).

Animals were randomly assigned into the following three groups (n = 10 per group, 5 males and 5 females): (1) control group; (2) single dose APAP (2500 mg/kg) group; (3) repeated dose APAP group (1000 mg/kg) group. Liver from each animal was divided into 5~6 different pieces, and they were paraffin-embedded. In total, about 200 liver sections were H&E-stained and digitalized into whole-slide images (WSIs) by slide scanner. For dataset establishment, images of necrosis, inflammation, infiltration, and portal triad were cropped and labeled from 7, 16, 30, and 132 whole-slide images. Thirty-two WSIs, which were not used for model training, were left to check the performance of the trained AI model.

Acetaminophen (APAP; A7085, 99.0% purity; Sigma-Aldrich, MO, USA) was administered orally to induce acute liver injury in 10-week-old SD rats using two dosing systems: a single dose of 2500 mg/kg or a 6-day repeated dose of 1000 mg/kg. Doses of APAP were chosen from previously published reports [22,23]. Immediately prior to administration, 2500 mg or 1000 mg of APAP was dissolved in 10 mL of sterile distilled water. Administration was performed at 10 mL/kg per dose. Sterile distilled water was administered as a vehicle control. The day of the starting dose was regarded as Day 1. Single-dosing animals and six-day repeated animals were sacrificed on Day 3 and Day 7, respectively. Liver tissues were collected in 10% formaldehyde. Hematoxylin and eosin (H&E) staining was performed as previously described [24].

### 2.2. Data Preparation

Whole-slide images (WSIs) of liver sections were scanned using a Panoramic 250 Flash III (3DHistech, Hungary) with a 20× objective and bright-field illumination. The scan resolution was 0.24 μm per pixel, and the images were saved as TIFF stripes with JPEG2000 image compression. The data preparation for segmentation of portal triad, necrosis, infiltration, and inflammation was performed as previously described [8]. Briefly, the 20×-magnified WSIs were cropped into 448 × 448 pixels of tile images, and all lesions were labeled using a VGG image annotator 2.0.1.0 (Visual Geometry Group, Oxford University, Oxford, UK). The annotated lesions were confirmed by an accredited toxicologic pathologist before the algorithm training was initiated. A total of 8,291 image tiles were obtained from 201 WSIs. The lesions identified on these images were labeled and used to train and test the Mask R-CNN algorithm. The train_test split function embedded in the scikit-learn package was used to split the annotated image tiles into the training, validation, and test data sets (ratio, 7:2:1, respectively). Data augmentation was conducted to improve the training dataset; this was performed eight times using a combination of image-augmenting techniques (reverse, rotation, and brightness). A total of 46,312 images were used for training, while 1659 and 843 images were used for validation and testing, respectively (Supplementary Material Table S1).

### 2.3. Generation of the Mask R-CNN Algorithm

**Figure 1:**
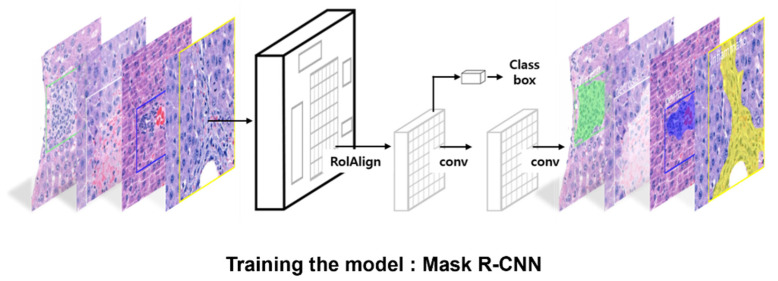
Schematic of the procedures for Mask R-CNN and segmentation of hepatic lesions.

All procedures related to algorithm training, including the data distribution, were performed as previously described in detail [8]. Briefly, the training was performed using an open-source framework for machine learning (Tensorflow 2.1.0 with a Keras 2.4.3 backend) powered by an NVIDIA RTX 3090 24G GPU. The Matterport Mask R-CNN 2.1 package (Sunnyvale, CA, USA) was used for training. The Mask R-CNN algorithm consisted of two stages: (1) the region proposal network (RPN), which proposed candidate object-bounding boxes; and (2) RoIAlign, which was used to extract features for the prediction of pixel-accurate masks. RoIAlign uses bi-linear interpolation to compute the exact values of the input features at four regularly sampled locations in each RoI bin and aggregates the results using max pooling. A schematic of the procedure is shown in Figure 1.

### 2.4. Model Training, Validation, and Testing for Acute Hepatocellular Injury

#### 2.4.1. Hyperparameters

A total of 48,814 images were used to train, validate, and test the model on lesions of acute hepatic injury in SD rats. The hyperparameters used during the training are described in Table 1. All configurations were set as the defaults defined by the Matterport package with the exception of the five parameters that were customized to fit the hepatic injury dataset. Four images were simultaneously analyzed using IMAGE_PER_GPU, and four GPUs were used during the training. The image size was determined as 448 × 448 by IMAGE_MAX_DIM and IMAGE_MIN_DIM according to the tile image size. The threshold of instance classification accuracy, DETECTION_MIN_CONFIDENCE, was adjusted to 0.5. Stochastic Gradient Descent (SGD) was selected as the optimizer.

#### 2.4.2. Loss

To calculate the training losses, the class (label), mask, and bounding box (bbox) losses observed during the training were serialized using the tf. Summary module and visualized using a tensorboard. To calculate class loss, we used the multi-class cross-entropy loss. Since the mask network uses the sigmoid to predict whether a given pixel belongs to the class, the mask loss was determined by binary cross-entropy. For the bounding box loss, we used a smooth L1 loss, which calculated the error between the prediction and ground truth. Finally, to determine the Mask R-CNN loss (total loss), we calculated the sum of the losses (i.e., the sparse softmax cross-entropy loss for the label, the smooth L1 loss for the bounding box, and the binary cross-entropy loss for the mask).

#### 2.4.3. Metrics for Model Performance

To verify model performance, we calculated the mean average precision (mAP), which is derived from the intersection of the union (IoU), precision, and recall values. The IoU value was calculated as previously described [8], and generally reflected the ratio of the area overlaid by the union of the predictions and the ground truth. The mAP value reflects the accuracy of the model; here, we used the transformed mAP, which takes on a value of 0 when an image is found to contain any misprediction. We used this transformation to perform a more detailed analysis of the error cases, investigate the causes of correct and incorrect predictions, and more strictly evaluate the model performance.

### 2.5. Model Performance Confirmation at the WSI Level

Thirty-two WSIs that were not used during the training were applied as the confirmation set. All WSIs were obtained from APAP-treated animals. These WSIs were scanned using a 20× objective and bright-field illumination. Before confirmation, the hepatic lesion (including the connective tissue) of each WSI was annotated by an accredited toxicologic pathologist as the ground truth to be compared with the prediction of the algorithm. After annotation, the area of the annotated region was calculated and transformed into a percentage of the liver-section area. WSI annotation and the annotated area calculation were conducted using Aperio Image Scope version 12.4.0 (Leica Biosystems, Richmond, IL, USA) and 20× magnification-scale images. Each magnified WSI was divided into 448 × 448 pixels of tile images, and each hepatic lesion was inferred by the trained algorithm. Following the prediction, the prediction mask-bearing cropped images were merged into a WSI. The prediction mask areas were calculated and compared to the annotated lesion by linear regression

## 3. Results

### 3.1. Training and Validation of the Mask R-CNN Algorithm for Acute Hepatic Injury Lesions

**Figure 2:**
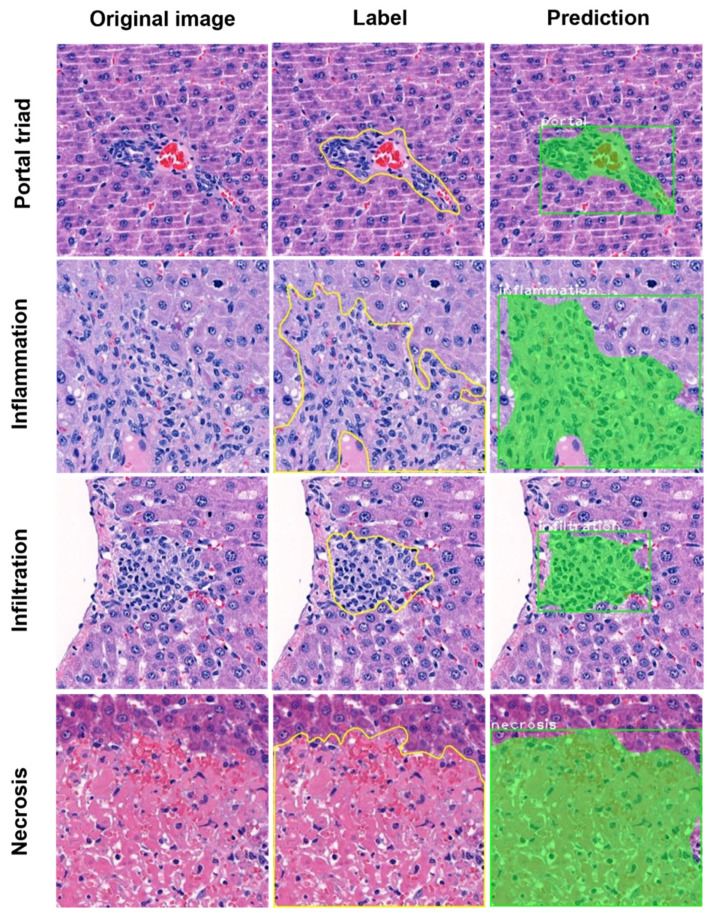
Representative segmentation for training and testing of hepatic lesions. The left panels show the original image tiles before labeling; the middle panels show labeled images for training of portal triad, necrosis, inflammation, and infiltration; the right panels show the predicted area for each lesion, as determined by Mask R-CNN.

To train the Mask R-CNN network for identification of acute hepatic injury lesion, a total of 46,312 annotated tile images, including the augmented samples, were used. Three hepatic lesion types, namely necrosis, inflammation, and infiltration, were trained for the identification of hepatic lesions. To improve the performance of the trained model, we also annotated portal triad, which we found could be confused with infiltration of mononuclear cell and histiocytes. Total losses, including class, mask, and bbox losses, decreased steadily during the training (Supplementary Material Figure S1). As shown in the right panels of Figure 2, the algorithm successfully distinguished between all trained lesions and normal liver cells in the image tiles. Moreover, the predicted hepatic lesions overlapped well with the labeled lesions, as shown in the middle and right panels of Figure 2.

During training and validation, we found that some of the detection results did not match the corresponding annotated lesion. Further assessment revealed that inflammatory findings in the annotation were incorrectly recognized as infiltration findings in the algorithm-based prediction (Supplementary Material Figure S2).

After training, we tested the model performance by generating mAP values for a total of 843 image tiles. The overall mAP was 96.44%, and the results obtained for portal triad, necrosis, inflammation, and infiltration were 95.10%, 100%, 96.35%, and 94.29%, respectively (Table 2). This model performance was considered to be outstanding, despite the confusion between inflammation- and infiltration-related lesions.

### 3.2. Model Performance Confirmation Using WSI

**Figure 3:**
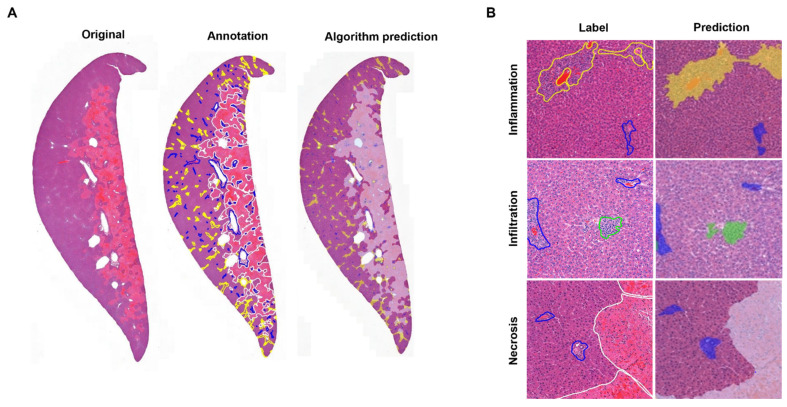
Comparison of the original image, annotated image, and algorithm-predicted image at the WSI level. (**A**) Original WSI, annotated result obtained from an accredited toxicologic pathologist, and algorithm detection results for hepatic lesion at 20× magnification. (**B**) Magnified WSI images including necrosis, inflammation, and infiltration lesions. Portal triad (blue), inflammation (yellow), infiltration (green), and necrosis (white) are shown as different colors.

To test the performance of our trained algorithm in a real-world setting, we tested its ability to predict hepatic lesions from 32 WSIs. The test was operated at 20× magnification. Portal triad (blue), necrosis (white), inflammation (yellow), and infiltration (green) were presented in different colors, as shown in Figure 3. The true annotated lesion was reported by the square micrometer (µm^2^), and the algorithm-estimated pixels were converted to the same units (µm^2^). Our results showed that the lesions annotated for portal triad, necrosis, inflammation, and infiltration were comparable to the images predicted by the model (Figure 3A). Using magnified WSI images, the AI algorithm successfully identified each lesion of necrosis, inflammation, and infiltration, as compared to the annotated lesions (Figure 3B).

**Figure 4:**
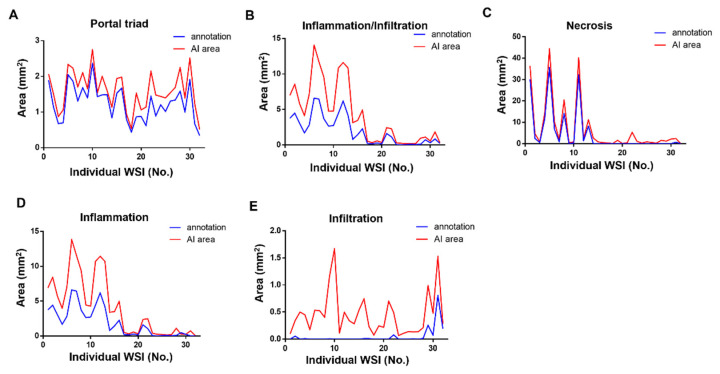
Comparison of the annotated and algorithm-predicted areas at the individual WSI level. Blue lines represent annotated area and red lines show algorithm-predicted area for portal triad (**A**), inflammation and infiltration together (**B**), necrosis (**C**), inflammation (**D**), and infiltration (**E**).

**Figure 5:**
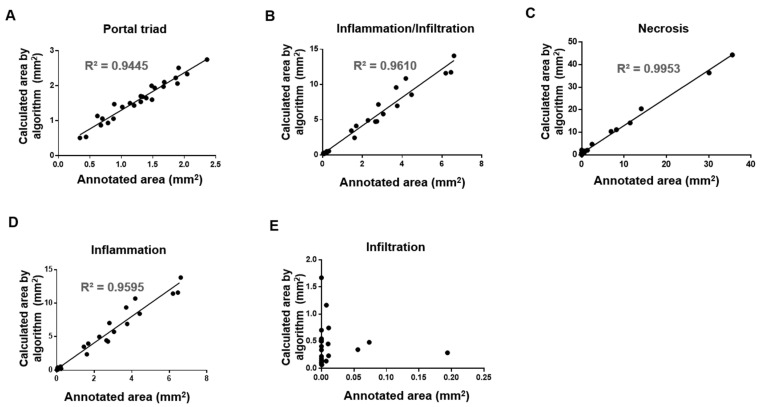
Correlations between annotated area and algorithm-predicted area. The areas annotated by an accredited toxicologic pathologist were compared by linear regression with those predicted by the established algorithm for portal triad (**A**), inflammation and infiltration together (**B**), necrosis (**C**), inflammation (**D**), and infiltration (**E**). The prediction was performed using 20× magnification-scale images.

In individual WSIs, the annotated lesion areas of necrosis, inflammation, and infiltration tended to be comparable to (albeit slightly larger than) those determined by the accredited toxicologic pathologist (Figure 4). Our combined evaluation revealed that the inflammation-plus-infiltration findings tended to show greater agreement with the annotated images, compared to either alone (Figure 4B,D,E). Correlations between the annotated and predicted lesions are shown in Figure 5. The predicted areas of portal triad and hepatic lesions showed very high correlations with the annotated dimensions; all R^2^ values were above 0.9, with the exception of that of infiltration (Figure 5).

## 4. Discussion

The development and integration of digital pathology and AI-based approaches to identify lesions from slide images can offer substantial advantages over traditional methods, such as by enabling spatial analysis while generating highly precise, unbiased, and consistent readouts that can be accessed remotely by pathologists [25]. In pre-clinical studies, CNN has been used to achieve quantitative and rapid assessment of pathological findings during drug discovery and development.

In the present study, we sought to use deep learning to implement an AI algorithm for the assessment of toxicological pathology in a non-clinical study. The model was built through training and validation for several hepatic lesions and used to predict one lesion. Going forward, training and testing with different hepatic lesions could be used to allow this algorithm to efficiently differentiate multiple hepatic lesions. The trained algorithm exhibited a total mAP of 96.44%, which is an outstanding result compared to those obtained in previous efforts to detect hepatic lesions [26,27]. Finally, we compared the annotation results assigned by an accredited toxicologic pathologist with the model prediction to evaluate model performance. The predicted lesions of portal triad, necrosis, and inflammation showed high correlations with the annotated lesions.

In several previous studies, deep-learning CNN-based algorithms were developed for detecting hepatic lesions. Heinemann et al. reported that automated deep-learning-based scores obtained using CNNs showed good agreement with the findings of a human pathologist [28]. In the CCl4- or CDAA-induced rodent models of non-alcoholic steatohepatitis (NASH), four histological features were scored (i.e., ballooning, inflammation, steatosis, and fibrosis). In another published report, a deep learning-based algorithm using CNN enabled the construction of a fully automated and accurate prediction model for scoring the stages of liver fibrosis [29]. However, although these previous studies evaluated the use of deep-learning algorithms for lesion scoring, this is the first work to use such an algorithm to predict the areas of hepatic lesions in an APAP-induced acute hepatic rat model.

APAP is widely used as an analgesic and antipyretic drug in the United States [30]. APAP-induced liver toxicity has been reported, and APAP is regarded as one of the most common pharmaceutical products capable of causing DILI. The mortality rate in APAP overdose patients is ~0.4%, which translates to 300 deaths annually in the United States [31]. New efforts to detect biomarkers of injured and necrotic hepatocytes seem promising, as it is important to identify APAP-induced acute liver injury patients at an early stage when lifesaving medical and surgical therapies can be provided. Going forward, AI approaches to predicting DILI could improve our understanding of the underlying mechanisms and our ability to anticipate hepatotoxicity for clinical applications [32].

We encountered several issues when developing our model to identify hepatic lesions. In the early stage of model establishment, portal triad was not included in the training process. However, our early results revealed that portal triad was often mistaken for inflammation or infiltration (Supplementary Material Figure S3). In the three-class model, the algorithm was trained in inflammation, infiltration, and necrosis. In this model, before training on the portal triad, confusion with the portal triad was observed with inflammatory or infiltration findings. This prompted us to include portal triad in further training, with the goal of increasing lesion recognition. Indeed, this addition, called the four-class model, improved the accuracy of lesion recognition (Supplementary Material Figure S3). We also found that some regions annotated as inflammation lesions were incorrectly predicted by the algorithm as infiltration lesions. This could lead the algorithm to over-estimate infiltration lesions relative to those identified by the accredited toxicologic pathologist. That said, there was a relatively low incidence of infiltration lesions in the studied model, so this error is not expected to significantly affect the overall prediction result. Indeed, our combined evaluation of the inflammatory and infiltration findings showed a greater agreement with the annotated findings. Simple mononuclear cell infiltration has been typically reported as inflammatory in the hepatic parenchymal tissues of normal rats [33]. It was slightly different from APAP-induced inflammation, which was characterized by histiocyte infiltration of an activated form with fluent cytoplasm around necrosis (Figure 2). A dataset of two different categories was prepared to prove whether two histological findings can be distinguished through the AI model. The dataset was divided into training, validation, and test set for model training and accuracy testing. The test result of the AI model for the infiltration was good and showed about 94% accuracy under the dataset environment. However, its test result in the WSI was not good and the correlation determinant (Figure 5E) was very low between the annotation and the result analyzed by AI model under the WSI, real world environment. The figures of infiltration and inflammation seemed confused because they have shared similar cellular components to some extent [34], and more diverse and complex figures would exist in the real world than in a dataset environment [35]. This categorization of infiltration and inflammation did not seem advisable in this study. The two findings are commonly inflammatory and were not confused with the other findings such as necrosis or portal triad. Therefore, it is thought to be more desirable to merge the values of infiltration and inflammation to evaluate the degree of inflammatory changes (Figure 5B). Finally, in WSI, connective tissue was often recognized as necrosis (Supplementary Material Figure S4). Since connective tissue was not included in our training for model establishment, WSIs including connective tissue were excluded from our evaluation of the model’s performance. Careful consideration and further study will be needed before this model algorithm can be translated to real-world use.

As result of prediction using WSIs, liver injury including trained lesions might be identified. However, due to the limitations of artificial intelligence, untrained patterns or images could not be accurately distinguished. If there are untrained lesion patterns, additional dataset training will be required to identify the lesion efficiently. The presented AI algorithm efficiently predicted trained lesions, such as inflammation/infiltration, necrosis and portal triad in acute hepatic injury of rats. Further analysis of patient samples will be required to validate for human application.

Modern advancements in digital pathology mean that large quantities of quality digitized data are available for algorithm developers, scientists, and pathologists worldwide. Collaborations across the fields of digital pathology, machine learning, and big data acquisition are paving the way to revolutionize medical pathology [36,37]. Within this setting, novel approaches have been employed for image analysis in digital pathology; an example of such an approach is deep learning, which involves multi-layered neural network architectures. Some deep-learning algorithms involve a slow and hierarchical process of learning data abstractions and representations between layers and can become computationally expensive when dealing with high-dimensional image data. This can be addressed by the use of convolutional neural networks (CNNs), which effectively scale up high-dimensional data [38]. In the present study, we applied the Mask R-CNN algorithm to evaluate hepatic lesions in an APAP-induced acute hepatic injury rat model. The study results suggested that this algorithm can be used to implement diagnosis and prediction of hepatic lesions. In the future, this strategy could potentially be deployed in clinical practice.

## Figures and Tables

**Table 1:** Hyperparameters used in Mask R-CNN training.

Hyperparameter	Value
IMAGES_PER_GPU	4
GPU_COUNT	4
STEPS_PER_EPOCH	10
IMAGE_MAX_DIM	448
IMAGE_MIN_DIM	448
LAYER_1	60
LAYER_2	120
LAYER_3	200
DETECTION_MIN_CONFIDENCE	0.9
LEARNING_RATE	0.001
LEARNING_MOMENTUM	0.9
WEIGHT_DECAY	0.0001
DETECTION_MAX_INSTANCES	100

**Table 2:** Mean average precision (mAP) in Mask R-CNN training.

	Portal Triad	Necrosis	Inflammation	Infiltration	Total
**mAP**	95.10%	100%	96.35%	94.29%	**96.44%**

## Data Availability

Not applicable.

## Supplementary Materials

The following supporting information can be downloaded at: https://www.mdpi.com/article/10.3390/diagnostics12061478/s1. Supplementary Material Table S1. Number of cropped tile images used for the model training, validation, and testing. Supplementary Material Figure S1. Training loss during model establish-ment. Supplementary Material Figure S2. Discrepancy in inflammation detection between annota-tion and algorithm prediction. Supplementary Material Figure S3. Comparison of portal triad de-tection between 3 class model and 4 class model. Supplementary Material Figure S4. Discrepancy in connective tissue detection between annotation and algorithm prediction.
