# Supplementary material for: Artificial Intelligence-Assisted Image Analysis of Acetaminophen-Induced Acute Hepatic Injury in Sprague-Dawley Rats
Source: Diagnostics (Basel). 2022 Jun 16;12(6):1478. doi: 10.3390/diagnostics12061478 (PMC9222125; doi:10.3390/diagnostics12061478)

Supplementary material Table S1. Number of cropped tile images used for the model training, validation, and testing. Numbers in brackets indicate data augmentation using a combination of image-augmenting techniques, such as reverse, rotation, and brightness.

Supplementary material Figure S1. Training loss during model establishment. Bbox (A), class (B), mask (C), and total (D) losses during training.

Supplementary material Figure S2. Discrepancy in inflammation detection between annotation and algorithm prediction. Yellow arrow indicates a region annotated as inflammation but incorrectly recognized as infiltration by the algorithm. Portal triad (blue), inflammation (yellow), infiltration (green), and necrosis (white) are shown as different colors.

Supplementary material Figure S3. Comparison of portal triad detection between 3 class model and 4 class model. In 3 class model, annotated portal triad was confused with inflammation or infiltration (upper panel). However, after training of portal triad, called 4 class model, improved the accuracy of portal triad recognition and confusion with other lesions. Portal triad (blue), inflammation (yellow), infiltration (green), and necrosis (white) are shown as different colors.

Supplementary material Figure S4. Discrepancy in connective tissue detection between annotation and algorithm prediction. Blue arrow indicates a region annotated as connective tissue but incorrectly recognized as necrosis by the algorithm. Portal triad (blue), inflammation (yellow), infiltration (green), and necrosis (white) are shown as different colors.

Supplementary material Table S1.

	Portal triad	Necrosis	Inflammation	Infiltration	Total
**Training**	1,397 (11,176)	1,470 (11,760)	1,462 (11,696)	1,460 (11,680)	5,789 (46,312)
**Validation**	399	420	421	419	1,659
**Test**	204	210	219	210	843
**Total**	11,779	12,390	12,336	12,309	48,814

Supplementary material Figure S1.

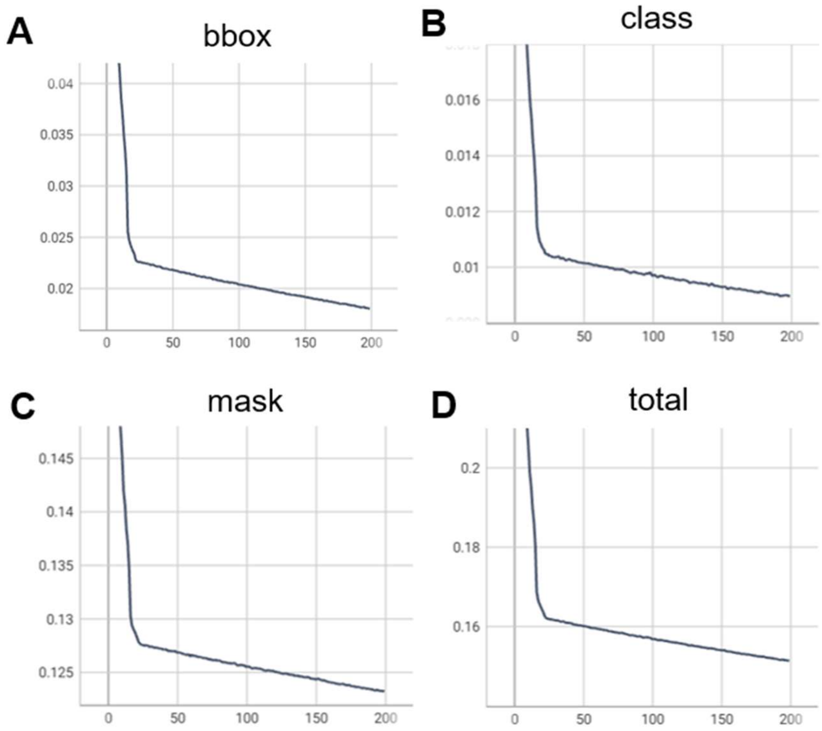

Supplementary material Figure S2.

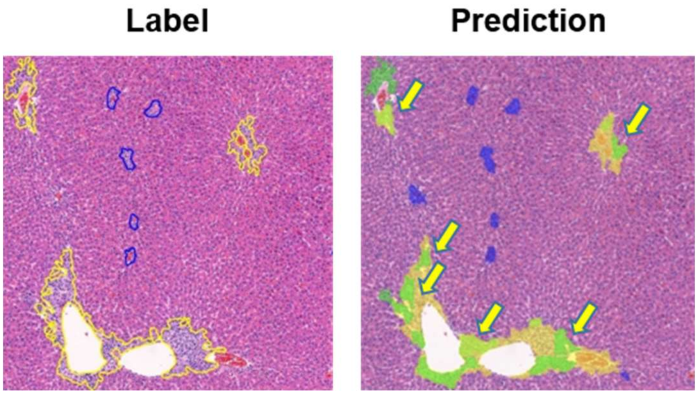

Supplementary material Figure S3.

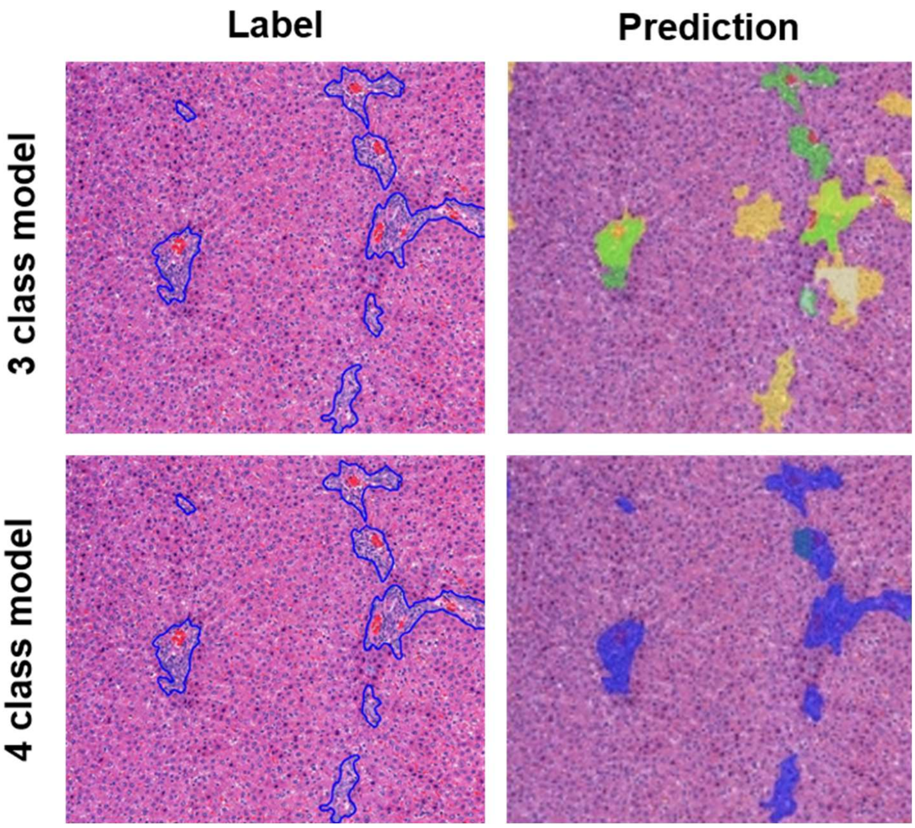

Supplementary material Figure S4.

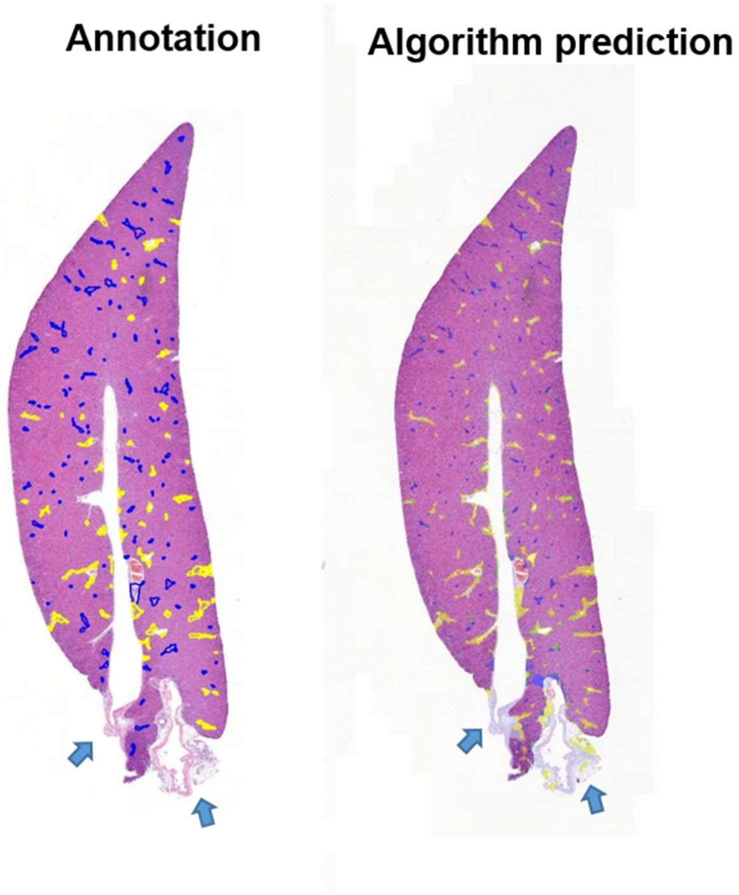

Supplement: Supplementary file 1 [file diagnostics-12-01478-s001.zip › diagnostics-1766020-supplementary.pdf]
